# Validation of a behavior observation form for geese reared in agroforestry systems

**DOI:** 10.1038/s41598-022-18070-6

**Published:** 2022-09-07

**Authors:** Alice Cartoni Mancinelli, Simona Mattioli, Laura Menchetti, Alessandro Dal Bosco, Diletta Chiattelli, Elisa Angelucci, Cesare Castellini

**Affiliations:** 1 Department of Agricultural, Food and Environmental Sciences, University of Perugia, 06100 Perugia, Italy; 2 Department of Veterinary Medicine and Animal Sciences, University of Milano, 26900 Lodi, Italy

**Keywords:** Behavioural ecology, Biodiversity, Ecosystem ecology

## Abstract

Agroforestry systems, which are based on the integration of trees and animals, represent a useful practice for implementing the “One Welfare” concept. Geese could adapt well to these systems due to their kinetic and grazing abilities. However, the lack of specific ethograms and animal-based measures have not yet allowed a deep assessment of their welfare and behavior. The aim of this study was to develop and validate a protocol to evaluate the behavior of geese reared in two agroforestry systems (i.e., apple orchard and vineyard). Thus, a behavior observation form (BOF) including a specific ethogram was proposed, and its interobserver reliability, content, criterion and construct validity were determined. Moreover, the influence of the time of day and type of agroforestry system on geese's behavior was also investigated. Agreement and principal component analyses, as well as the comparison between data collected through direct observation by the BOF and indirect observation by a computerized system, supported the reliability and validity of the proposed protocol. While the BOF also highlighted differences in the behavior expressed by the geese according to the time of day and the environmental context, both the vineyard and apple orchard systems seem to meet their biological and behavioral needs.

## Introduction

Currently, the social debate about animal welfare has achieved significant prominence at a global level^1^, stimulating scientific research to develop methods for improving animal husbandry. The increasing need for friendlier farming methods has played an important role in the European Citizens’ Initiative^2^ in demanding the end of cages for farmed animals. Thus, the impact of animal welfare has achieved relevant importance in society and scientific studies through the “One Welfare” concept. This idea, derived from the "One Health" concept, aims to highlight the numerous links among animal welfare, human welfare, and environmental sustainability^3,4^. In general, animals raised in extensive systems can exhibit all of their behavioral repertoire, thereby showing an increase in their welfare status. However, the conversion of the current intensive livestock production into extensive systems would require a high land use with the consequent effect on environmental sustainability^5^. Thus, it is important to pay attention to those production systems that improve both animal welfare and environmental protection. For instance, the combination of perennial crops (such as orchards, vineyards or olive groves) and animals in the same area could be a good sustainable practice. This strategy is recognized as an agroforestry system and is characterized by different levels of integration, such as crops and trees, crops and animals, and trees and animals^6^.

In the context of agroforestry practices, the goose represents a very interesting species due to its grazing ability and the use of high-fiber feed. Moreover, geese do not require impactful and expensive housing systems when reared outdoors^7^; thus, they fit well with the aim of improving environmental sustainability through agricultural integrated practices. Massaccesi et al.^8^ showed that geese reared in vineyards improve the efficiency of the soil microbial biomass and, through grass intake, remove the excessive copper needed to protect grapes from the ground. The grazing activity of geese naturally controls weeds by reducing the use of chemical and mechanical means. They also provide organic matter and natural fertilizers to the soil through their droppings^9^. Moreover, access to pasture could also modify the meat quality of geese. Cartoni Mancinelli et al.^10^ found a reduction in fat content and an increase in the development of drumstick muscle in the meat of geese reared in a vineyard. These studies confirm that geese reared in an agroforestry system are environmentally sustainable and positively influence meat characteristics. However, animal welfare and behavior have not yet been deeply investigated.

Overall, there are few specific animal-based measures (ABMs) for the goose; in particular, there is a lack of tools to evaluate the welfare principle of Appropriate behavior. Recently, Tremolada et al.^11^ proposed a feasible and effective welfare assessment protocol for geese, including a shortlist of ABMs, such as plumage condition, wings and ability to walk assessment, and a handling test. However, only a few studies have reported a complete behavioral ethogram for geese. In addition, the protocol of Tremolada and colleagues^11^ was developed for commercial production, while the agroforestry system is a peculiar scenario, and its impact on animal welfare has not been investigated. As suggested by the European Food Safety Authority (EFSA), welfare assessment must fit the exposure scenario, including housing, nutrition, farming and management procedures, and ABMs should be “fit for purpose”, i.e., addressed to specific objectives of the assessment for that particular species and category of the animal at that time^12,13^.

Although geese are considered to have been the first domesticated poultry species, most research on welfare, farming systems, and veterinary care has focused on chickens. However, there are many differences in terms of behavior between geese and modern chicken genotypes. Geese belonging to the Anatidae family are waterfowl animals; they present a higher body mass than chickens and have other peculiar characteristics, such as webbed paws, wide beak with transversal gills to filter the water for food search, and a gland secretion fat necessary to waterproof the plumage. Therefore, many ABMs used in chickens are not suitable for geese, especially with regard to behavioral repertoire assessment; thus, a specific ethogram needs to be developed. Finally, the ABMs included in the welfare principle of Appropriate behavior should be feasible, reliable, and validated. Thus, the collection method should not be expensive in terms of human, economic, and time resources^12^, and ABMs should be validated through rigorous statistical approaches^12,14–16^.

This study aimed to develop and validate a feasible protocol to evaluate the behavior of geese reared in agroforestry systems. Specifically, a behavior observation form, including a list of behavioral variables, was proposed, and its interobserver reliability, as well as content, criterion and construct validity, were determined. As part of the construct validity analyses, the influence of the time of day and type of agroforestry system (apple orchard and vineyard) on geese's behavior was also investigated.

## Results

### Descriptive statistics and interobserver reliability

Table 1 shows the mean frequencies of each behavioral variable recorded by the two observers and the significance of their differences, while related Intraclass correlation coefficients (ICCs) are reported in Table 2. Ten variables showed good or excellent interobserver reliability (ICC ≥ 0.60). Poor or fair interobserver reliability (ICC < 0.60) was found for 8 out of 18 variables, particularly for rarer behaviors such as allo-grooming, squawking, getting wet, neck up, neck forward, panting, stretching, and wagging tail. All of these behaviors had a mean frequency < 1 (e.g., Table 1), and many of these had never been noticed by Observer 2. In general, the frequencies of the behaviors recorded by Observer 2 were lower than those recorded by Observer 1, and significant differences were found for 4 variables (i.e., foraging, resting, getting wet, and feeding). The overall interobserver reliability of the BOF was, however, excellent (ICC > 0.75).

**Table 1:** Mean and standard deviation (s.d.) of the frequencies recorded by the two observers for each behavioral variable.

Behavior	Observer 1 (main observer)		Observer 2		*p* value *
	Mean	s.d	Mean	s.d	
Walking	3.35	3.725	3.38	2.667	0.992
Resting	2.50	1.062	1.98	0.920	** < 0.001**
Roosting	1.43	2.500	1.50	2.522	0.499
Foraging	3.93	4.582	2.53	3.146	** < 0.001**
Feeding	0.18	0.501	0.05	0.221	**0.025**
Drinking	1.23	1.527	1.05	1.260	0.275
Getting wet	0.78	2.166	0.13	0.335	**0.024**
Self-grooming	1.90	2.560	1.85	2.131	0.815
Aggression	0.03	0.158	0.03	0.158	1.000
Flapping wings	1.18	1.866	0.85	1.312	0.103
Allo-grooming	0.10	0.496	0.00	0.000	0.180
Squawking	0.05	0.221	0.00	0.000	0.157
Wagging tail	0.03	0.158	0.00	0.000	0.317
Neck forward	0.28	0.452	0.15	0.362	0.132
Neck up	0.03	0.158	0.00	0.000	0.317
Shaking head	0.18	0.501	0.15	0.427	0.655
Stretching	0.03	0.158	0.00	0.000	0.317
Panting	0.03	0.158	0.00	0.000	0.317

Significant values are in bold.

* Wilcoxon signed rank test.

**Table 2:** Interobserver reliability. Intraclass correlation coefficients (ICCs) of the behavioral variables assessed by two observers. Each ICC is followed by its 95% confidence interval (CI) and by the *p* value of the *F* test.

Behavior	ICC	95% CI		*p* value*
		Lower bound	Upper bound	
Walking	0.896	0.812	0.944	< 0.001
Resting	0.792	0.641	0.885	< 0.001
Roosting	0.962	0.929	0.980	< 0.001
Foraging	0.897	0.814	0.944	< 0.001
Feeding	0.625	0.393	0.783	< 0.001
Drinking	0.701	0.500	0.830	< 0.001
Getting wet	**0.151**	− 0.165	0.439	0.173
Self-grooming	0.700	0.499	0.829	< 0.001
Aggression	1.000	1.000	1.000	–
Flapping wings	0.681	0.471	0.817	< 0.001
Allo-grooming	**0.000**	− 0.308	0.308	0.500
Squawking	**0.000**	− 0.308	0.308	0.500
Wagging tail	**0.000**	− 0.308	0.308	0.500
Neck forward	**0.207**	− 0.109	0.484	0.098
Neck up	**0.000**	− 0.308	0.308	0.500
Shaking head	0.705	0.507	0.832	< 0.001
Stretching	**0.000**	− 0.308	0.308	0.500
Panting	**0.000**	− 0.308	0.308	0.500
**Overall**	0.859	0.838	0.877	< 0.001

Poor or fair interobserver reliability (ICC < 0.60) is shown in bold.

*F test for ICC.

–Not computed as there was perfect agreement.

### Validity

Table 3 shows the results of the principal component analyses (PCA) investigating whether the variables included in the BOF could measure the broad behavioral traits of geese. Resting, feeding, getting wet, panting, allo-grooming, and shaking were not included in the PCA, as they showed low communalities and/or correlations. Four principal components (PCs) were extracted that explained more than 75% of the variance.

**Table 3:** Loadings of behavioral variables of factors extracted with the principal component analysis.

Item	Component			
	PC1- Activity	PC2- Social interaction	PC3- Comfort and body care	PC4- Social avoidance
Drinking	**0.919**	0.062	0.231	0.085
Walking	**0.913**	0.074	− 0.256	− 0.060
Foraging	**0.903**	0.169	− 0.117	− 0.092
Roosting	− **0.635**	0.146	**0.585**	0.169
Neck up	− 0.046	**0.848**	0.238	− 0.068
Squawking	0.121	**0.779**	0.197	0.305
Wagging tail	0.042	**0.759**	− 0.090	0.103
Aggression	0.173	**0.626**	− 0.305	0.116
Self-grooming	0.127	− 0.067	**0.925**	0.196
Flapping wings	− 0.230	0.075	**0.821**	0.007
Neck forward	− 0.014	0.030	0.044	**0.930**
Stretching	− 0.124	0.362	0.187	**0.755**
% Variance explained	29.0	25.2	14.4	9.8
Cumulative % variance explained	78.4			
Cronbach’s alpha	0.890	0.692	0.780	0.706

Loadings with an absolute value greater than 0.5 are in bold.

The first PC (Activity) accounted for more than 25% of the variance and had excellent reliability (i.e., Cronbach’s alpha > 0.7). It was bipolar and included variables indicating locomotor and exploratory activity with opposite signs to roosting. A high score for this PC could indicate dynamism, while a low score could indicate rest and relaxation.

The reliability of PC2 (Social interaction) was lower than that of PC1 and bordered acceptability. It included variables of communicative behaviors, but there were indicators of both positive and negative interactions. A high score for PC2 could indicate a high degree of social interactions, including aggressive behaviors.

The third PC (Comfort and body care) had good reliability and was clearly defined by comfort and cleanliness behaviors, including variables indicating self-care.

Finally, the highest loading of the PC4 (Social avoidance) was found for hostile behavior, i.e., neck forward. It was positively associated with stretching, and the two items showed acceptable reliability.

Table 4 shows the strength of association between the frequencies assessed through the direct (i.e., BOF) and indirect (i.e., gold standard, Noldus) observations. The overall correlation indicated excellent criterion validity (ρ = 0.893); only 2 of the 18 behavioral variables showed poor (ρ < │0.3│) correlations. The frequencies noted using the Noldus were, however, higher than those collected in the field for most of the behavioral variables (e.g., Supplementary Table S1).

**Table 4:** Criterion validity. Spearman rank correlation coefficient (ρ) measuring the association between the frequencies obtained by the behavior observation form (BOF) in the field and the ‘gold standard’ measure (i.e., Noldus).

Behavior	Spearman rank correlation coefficient ρ	*p* value
Walking	0.990	< 0.001
Resting	0.843	< 0.001
Roosting	0.999	< 0.001
Foraging	0.988	< 0.001
Feeding	0.998	< 0.001
Drinking	0.990	< 0.001
Getting wet	1.000	–
Self-grooming	0.996	< 0.001
Aggression	1.000	–
Flapping wings	0.993	< 0.001
Allo-grooming	1.000	–
Squawking	**0.125**	0.441
Wagging tail	**0.270**	0.093
Neck forward	0.990	< 0.001
Neck up	0.339	0.032
Shaking head	0.530	< 0.001
Stretching	0.310	0.052
Panting	1.000	–
Overall	0.893	< 0.001

Poor correlation coefficients (ρ < │0.3│) are in bold.

–Not computed as there was perfect association of ranks.

**Figure 1:**
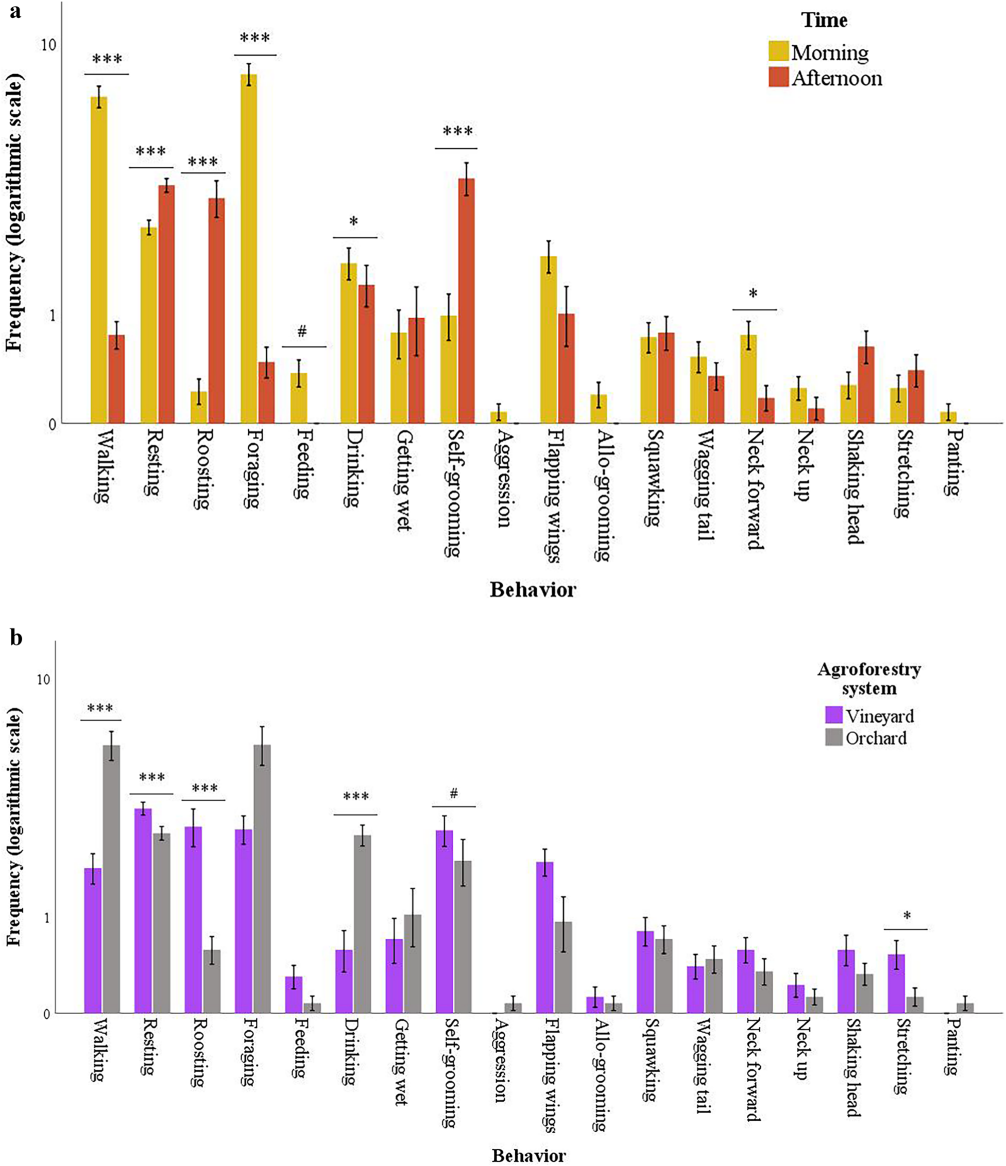
Geese's behavior. Absolute frequencies of behaviors of geese collected by focal subgroup sampling according to (**a**) the time of day and (**b**) the agroforestry system (*N* = 40, 30-min session). The values are raw data (mean ± standard error), but the differences indicated by the asterisks (***p ≤ 0.001, *p ≤ 0.05, ^#^*p* < 0.1) result from models including the geese as subjects and time as a within-subject effect.

The construct validity and responsiveness of the BOF were verified through the analysis of the behavioral changes of geese over time that were reared in two different agroforestry systems. The exact p values for time and system effects are detailed in Supplementary Table S2, while Fig. 1a compares each behavior collected in the morning and in the afternoon, regardless of the agroforestry system. The observed frequencies of walking (*p* < 0.001), foraging (*p* < 0.001), drinking (*p* = 0.048), neck forward (*p* = 0.021), and feeding (as trend, *p* = 0.076) were greater in the morning than in the afternoon, while those of resting (*p* < 0.001), roosting (*p* < 0.001), and self-grooming (*p* < 0.001) were greater in the afternoon. Differences in behavior between morning and afternoon confirm the responsiveness over time of the BOF.

Figure 1b compares the behavior of geese reared in the vineyard and the orchard, regardless of the time of day. Geese kept in the vineyard showed lower frequencies for walking (*p* < 0.001) and drinking (*p* < 0.001) and higher frequencies for resting (*p* < 0.001), roosting (*p* < 0.001), and stretching (*p* = 0.036) than those kept in the orchard. A trend was also found for self-grooming (*p* = 0.099), which was higher in the vineyard geese than in the orchard geese.

**Figure 2:**
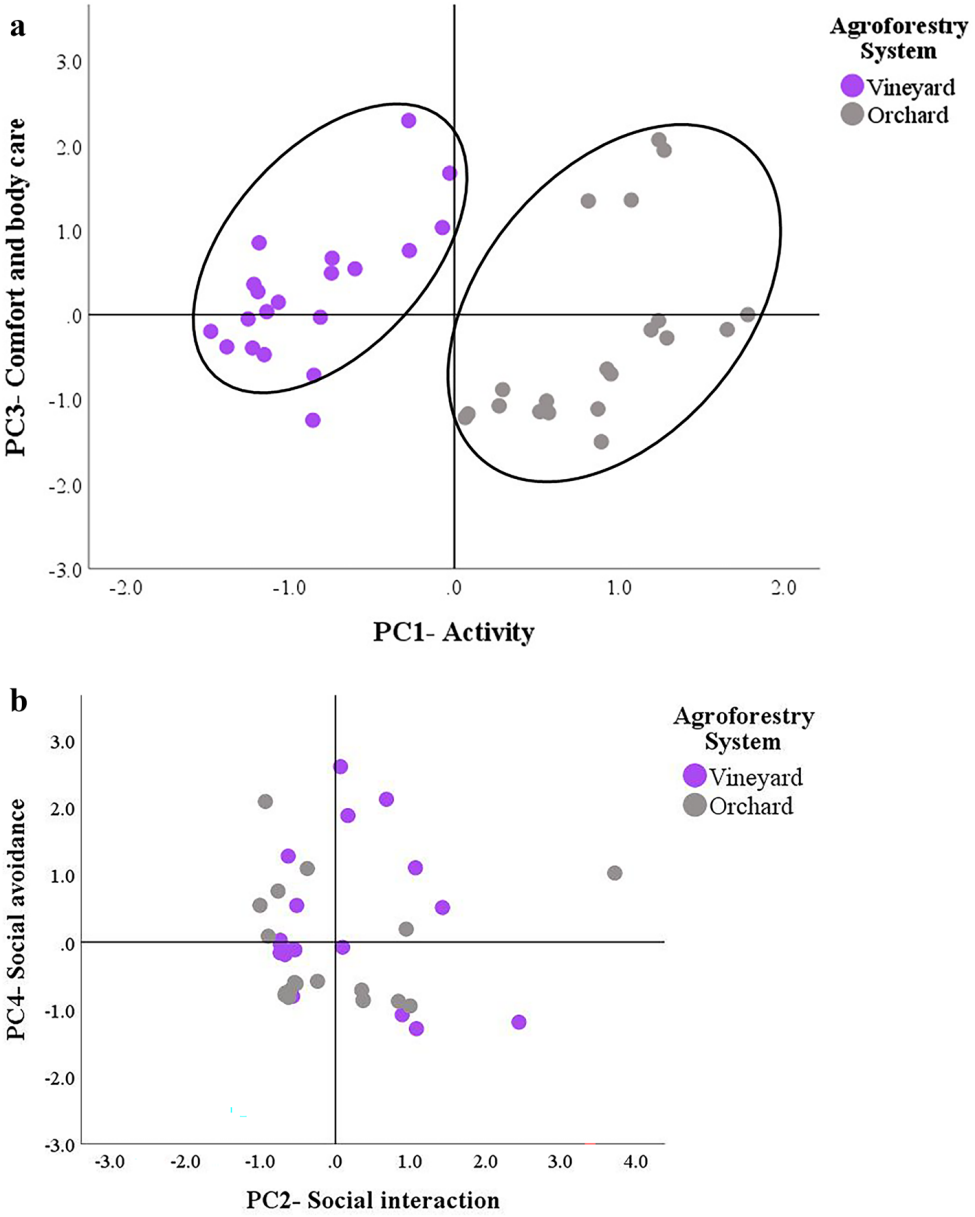
Principal component analysis score plots. Score plots of the dimensions related to (**a**) locomotor-relaxation activity (e.g., PC1 and PC3) and (**b**) sociability (e.g., PC2 and PC4) according to the agroforestry system.

Figure 2 shows the score plots for the four dimensions extracted by the PCA according to the agroforestry system. The arrangement of the PC scores confirms that the major differences between the two agroforestry systems concerned the locomotor and relaxation dimensions. In particular, the PC1-Activity separated the data into two clusters (Fig. 2a); the right cluster, indicating greater locomotor and exploratory activity, only contained geese kept in the orchard, while the left cluster included those kept in the vineyard. Except for an outlier, the geese kept in the vineyard had either positive or not strongly negative scores for PC3, suggesting a higher frequency of comfort behaviors. In contrast, no well-defined clusters could be found in the dimensions indicating the sociability of the geese (e.g., PC2 and PC4, Fig. 2b).

Differences between groups detected by direct observations support the construct validity of the behavior observation forms.

## Discussion

This study proposed a protocol to evaluate the behavior of geese reared outdoors in agroforestry systems. A data collection form (i.e., BOF) was developed and validated both in relation to its reliability and its validity. In this context, moreover, ABMs useful for a welfare assessment protocol could be defined, and changes in the behavior of geese due to daily time and environmental context could be identified.

Behavioral observations, based on the capture of the major changes in an animal's body language^17^, are used daily in the assessment of animal health and welfare. Body language is a type of dynamic expression of the interactions among conspecifics or between animals and their environment. Behavioral changes can happen quickly or as subtle shifts not easily detectable^18^. Indeed, especially in the case of direct observation in the field, it becomes difficult to identify each behavioral variation. Furthermore, the on-farm use of the BOF proposed in the present study involved focal subgroup sampling, as ten geese were simultaneously observed, which may increase the difficulties. Indirect observation by videos, which allow the review of a certain action several times and the focal-animal approach, is a useful tool to partially overcome these issues and thus improve the accuracy of observation. The validation process of the BOF adopted in this study, therefore, included the definition of both its interobserver reliability and correlation with indirect observations.

In this study, the direct observations in the field were performed by both an expert (i.e., main observer) and an inexperienced trained observer. As expected, the main observer was able to detect a higher frequency of behaviors, especially the rarer ones. For example, the inexperienced observer did not report any examples of allo-grooming, squawking, wagging tail, stretching, or panting behavior. However, the two observers showed excellent interobserver reliability (ICC > 0.75). Major agreements were found for walking, roosting, and foraging. Accordingly, several studies have shown that observers with little experience can also provide a valuable contribution in observational research^19,20^. Overall, these results support the reliability of the BOF even if the observer's experience helps him or her to better grasp rarer behaviors, as these behaviors could play an important role as welfare indicators.

In the last two decades, important technological developments have occurred in the livestock sector. The use of sensors, cameras, and other devices can generate objective information about individual behavior, thereby allowing its evaluation in large observation areas and for large groups of animals and resulting in the better detection of natural animal behavior. Thus, in our study, the data collected by a video recording system (Noldus XT) were used as a gold standard measure to define the criterion validity of the BOF. Our results indicated excellent agreement between direct and indirect observations, supporting the BOF criterion validity. A poor correlation was only found for 2 variables (i.e., squawking and wagging tail), which were more difficult to collect by direct observation. The use of the BOF involved the simultaneous observation of 10 animals, but the geese had a synchronized behavior and moved in groups within the grazing area. This greatly facilitated focal subgroup sampling, allowed all animals to always be under observation, and could explain the high correlation between the two observation methods. However, the comparison between the observations collected in the field by the main observer and those recorded using the computerized system confirmed the greater accuracy of the latter. The analysis of the video in continuous with the use of some tools, such as the zoom or slow-motion functions, and the focal-animal sampling provided an easier identification of some behaviors and, in general, greater accuracy. Due to its nonintrusive approach, video recording has become a common practice for behavior assessment^21^, but it can be expensive and time-consuming. On the other hand, direct observations made by the BOF were valid and less expensive, suggesting that it could be a feasible tool with which to evaluate the welfare principle of Appropriate behavior. As recommended for welfare assessment protocols^22^, the BOF ethogram included indicators of both positive and negative states; however, it would be necessary to integrate it with behavioral tests and other ABMs evaluating the human-animal relationship.

As mentioned above, there is no standardized geese behavior ethogram. Thus, to verify the content validity of the BOF, its behavior variables were analyzed through a PCA. The 4 extracted PCs could represent the broad behavioral dimensions of geese. In particular, the geese’s activity reported in PC1 was characterized by locomotor, foraging, and exploratory behaviors, with opposite signs with respect to roosting. The positive correlation between explorative and grazing activities and their negative correlation with static behaviors has been widely demonstrated in chickens. Chicken genotypes characterized by low exploratory aptitude exhibited low kinetic behaviors but a high frequency of roost and rest behaviors^23^. Göransson et al.^24^ showed that 50% of the observed birds exhibited sitting behavior, whereas less than 10% performed foraging activity.

PC2 included all the variables that characterized the geese's social aspects, including both positive and negative interactions. Usually, greylag geese live in a large flock because the offspring remain with their parents for an entire year. Such groups are characterized by complex relationships based on social interactions^25^. The formation of a group is characterized by agonistic behaviors such as fighting, pecking, and threatening, as well as submissive behaviors such as avoiding contact, crouching, and escaping^26^ to establish a hierarchical order. After this phase, a tolerance status develops, and birds maintain their social interactions through the use of body postures and vocalizations. Accordingly, the variables reported in PC2 were related not only to aggressive behaviors but also to geese’s vocalization and posture, which probably helped to maintain flock stability. Therefore, a higher PC2 score could indicate the need to establish and maintain a hierarchical order within the group, resulting in high social interactions.

PC3 reported comfort and body care behaviors. The opportunity to spend a lot of time on body care, which should also include access to water for bathing, is of paramount importance with regard to fulfilling the biological requirements of geese^27^. Thus, a higher loading of this PC means that animals showed a good degree of both welfare and adaptability. In our study, a high frequency of self-cleaning and wing flapping behaviors was recorded, and the geese often took advantage of the water tub. In contrast, a very low frequency of aggression behaviors was observed, suggesting that the groups of geese were quite stable and that the animals felt safe in the environment in which they were rear. These findings confirm that agroforestry has a favorable impact on bird welfare by allowing the display of the full range of behavior, improving the animals' comfort^28^.

PC4 was mainly represented by the neck forward behavior. This position only occasionally represents an attack behavior and is not utilized during the establishment of hierarchical order but when it is necessary to maintain and reinforce the order inside the group. Furthermore, a goose that assumes this posture often does so while continuing another activity^29^. The neck forward behavior was positively associated with the stretching behavior. Stretching is usually categorized as a comfort behavior for broilers^30^, but it could also be used when the animal needs to relax stress-related tension in their muscles^31,32^ or as an adaptive strategy for dealing with unknown contexts^33^. Neck forward and stretching were eventually considered social avoidance behaviors, although they could be ambivalent and thus require further study, case-by-case assessment, and perhaps a better description in the ethogram.

Finally, some interesting results emerged regarding the comparison of geese's behavior during the morning and afternoon and between the two different agroforestry systems. In particular, geese showed a higher frequency of active behaviors such as walking, foraging, drinking, neck forward, and feeding during the morning compared to the afternoon. All of these behaviors suggest that geese concentrate their grazing and exploration activities during the morning. When and where to move is crucial for the food search and to avoid both predators and adverse climate conditions^34^. Cartoni Mancinelli et al.^35^ included exploratory attitude, walking, and eating grass activities in a multifactorial score as important parameters to consider to evaluate the adaptability of different organically reared chicken genotypes. Thus, exploratory and kinetic behaviors are fundamental, especially in animals reared outdoors. Moreover, the positive correlation between walking and grazing behaviors is widely known^36,37^. In contrast, during the afternoon, geese showed higher frequencies of static behaviors such as resting, roosting, and self-grooming, suggesting that geese are more dedicated to comfort and body care activities during this time. These trials were performed in the hottest season; thus, the geese's behavioral differences during the day could also depend on the fact that animals preferred to carry out active behaviors during the cooler hours (morning), while in the hottest hours (afternoon), they engaged in static activities. Active behaviors cause an increase in metabolism and body temperature^38^, whereas static behavior, such as roosting, is considered adaptative behavior to promote heat dissipation^31,39^.

This could also explain why higher frequencies of walking and foraging and lower frequencies of static behaviors were found in the orchard system than in the vineyard system. Studies carried out on chickens have reported that, among different pasture enrichments, the presence of trees promotes walking animal activity compared with crop inclusion^40,41^. The cover provided by trees made the animals feel protected from predators and provided shade during the hottest part of the day^40^, thereby stimulating the animals to explore all the available space in the pen. Accordingly, geese reared in the apple orchard ingested more grass than those reared in a vineyard^36^. However, there were no differences between the two systems for social behaviors. Moreover, the highest frequency of roosting and self-cleanliness behaviors was recorded in the vineyard, suggesting that this space offered a comfortable environment and that both systems seem respectful of the biological needs and welfare of the geese.

The behavioral assessment protocol proposed in this study involving the BOF ethogram was feasible, low-cost, fast, and responsive both over time and between housing systems. It could thus be used for the assessment of Appropriate behavior in a welfare assessment protocol for geese reared in outdoor or free-range systems, although it lacks indicators of the human-animal relationship, such as avoidance distance or handling tests; such a scoring system should be developed. Regarding the specific behaviors in the two agroforestry systems, it should also be noted that they are difficult to generalize, as the characteristics of the plants, the environment, and management could have influenced these traits. Specifically, the behaviors could have been affected by the temperatures; therefore, further trials at different altitudes, seasons (i.e., autumn and winter), and climate are necessary for external validation.

## Conclusion

The present study developed for the first time a complete goose ethogram that could be used in two different ways, namely, direct observation in the field by a behavioral observation form (i.e., BOF) and indirect observation by videotapes and computerized systems. Direct observation can be less expensive and feasible tool that could be included in a goose welfare assessment protocol, although indirect observation is more accurate. This study, however, has confirmed the reliability and validity of the BOF, as it has shown herein excellent interobserver agreement and results in terms of content, criterion, and construct validity.

The validated BOF was then applied to evaluate the geese’s behaviors in different agroforestry systems two times a day. Geese are active animals that, nevertheless, dedicate a large amount of time to body caring and show remarkable synchronization of behaviors. During the morning, they spend time grazing and exploring the pen, while during the afternoon, they prefer to engage in comfort and body care activities. Different behaviors also emerged between the two agroforestry systems, with the highest active behaviors observed in the apple orchard and the highest expression of comfort behaviors detected in the vineyard system. These findings suggest that the apple trees provide geese with a good microclimate, but the vineyard also offers them a comfortable environment. Thus, both systems seem respectful of their biological needs and welfare.

## Methods

### Housing, animals, and management

**Figure 3:**
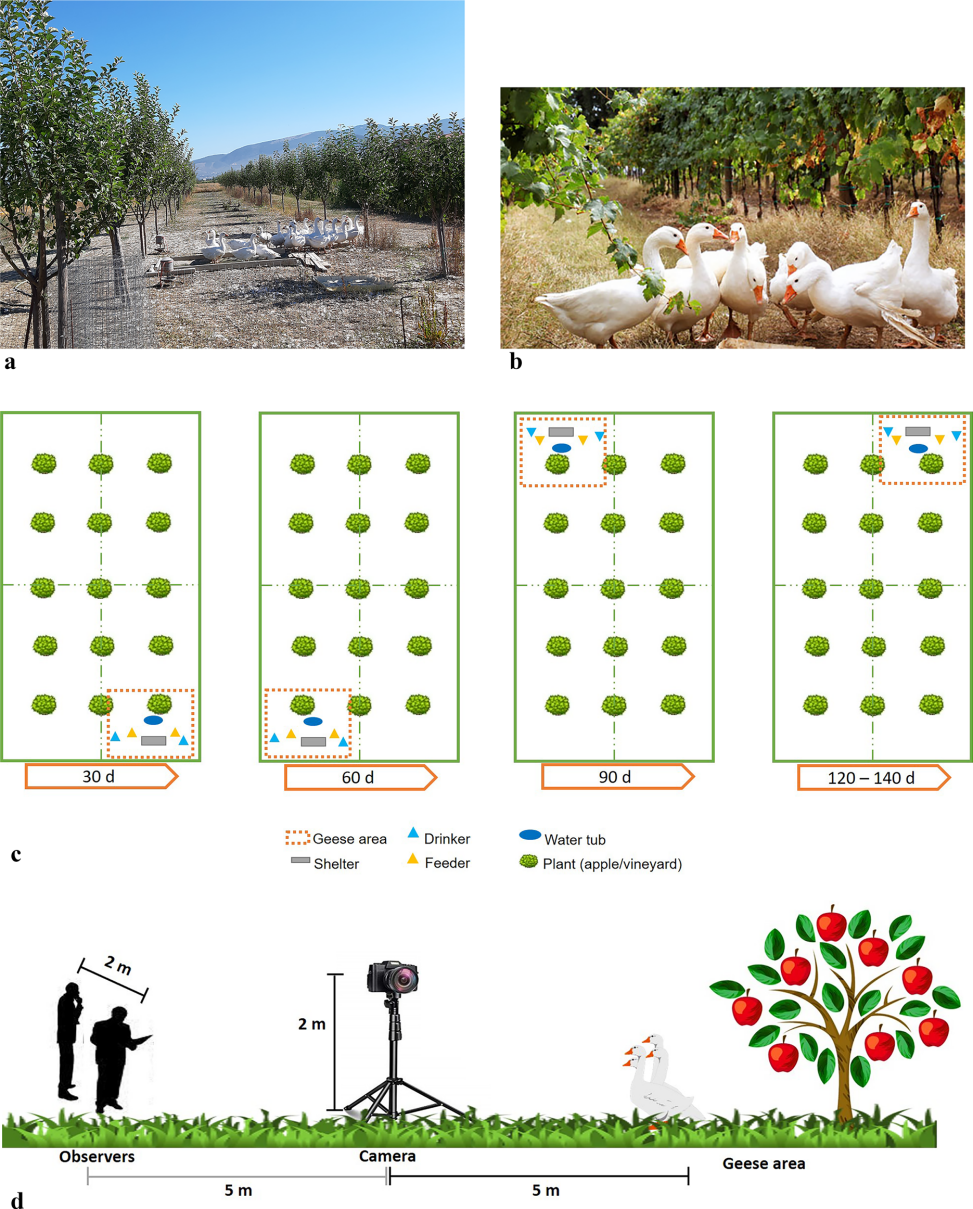
Housing, animals, and management. Geese in the (**a**) apple orchard and (**b**) vineyard. (**c**) Graphical representation of the “geese area” displacement. The mobile shelter with the other facilities was moved within the pen every 30 days. (**d**) Graphical representation of the study site indicating the position of the camera and observers in relation to the geese area.

Animals were reared from April to August 2019 in two organic farms located in the same area of Perugia (Umbria, central Italy, 42°59′19.78″ N–12°33′00.41″ E) at approximately 250 m a.s.l. Each farm was characterized by a different agroforestry system: apple orchard (AO, Fig. 3a) and vineyard (V, Fig. 3b).

The characteristics of the area were described in our previous study^8^. Briefly, both the V and AO were organic; accordingly, the only treatment allowed was the spraying of Cu-based fungicide [copper oxychloride, Cu2(OH)3Cl]. The apple orchard exhibited a planting distance of 5 × 8 m, whereas the vineyard showed a planting layout of 0.8 × 1 m. The area was characterized by a continental climate with a mean annual temperature of 13.8 °C, whereas the mean annual precipitation was 859 mm. The temperatures and humidity recorded during the behavioral observation period (from June to August 2019) are shown in Supplementary Table S3.

One hundred one-day-old female Romagnola geese were divided into two groups; 50 geese were housed in the AO (2 replicates of 25 geese each; Fig. 3a), while the other 50 were housed in the V (2 replicates of 25 geese each; Fig. 3b). The geese were raised in the same conditions, and a specific rearing technique was applied to combine the vegetable production (grapes and apples) with the behavioral characteristics of the geese. At 20 days of age, the geese were kept in a shelter with the following environmentally controlled parameters: temperature from 25 to 32 °C, relative humidity from 65 to 75%, and indoor density of 5 geese/m2. At 21 days of age, the geese were allowed access to the outdoor area during the day (total dimension of pen = 7,500 m2) with a density of 300 m2/goose. To prevent geese from causing damage to plant production or annoyance during normal agricultural practices, free access to pens coincided with the spring season. Indeed, during this period, the branches of the vineyard are sufficiently developed (> 30 cm in length), while the pruning activity has been completed in the apple orchard.

Moreover, the shelter designed for the geese was transportable and moved periodically within each pen. Among the avian species, geese indeed present remarkable grazing behavior and a long rearing cycle (5 months). Moreover, the webbed paws of geese tend to compact the soil, especially in the areas where they stay for a long time. Such an area, comprising the mobile shelter, a water tub, 2 feeders, and 2 drinkers both inside and outside the shelter (4 in total), was identified as the “geese area”. Therefore, geese require relevant amounts of outdoor space with accurate management. To ensure low animal pressure both on the pasture (grass growth) and on vegetable production, the pen was divided into 4 subpens. Every 30 days, both the animals and the “geese area” were moved from one subpen to another (Fig. 3c). Considering that for the first 20 d of life, geese were kept inside the shelter, the geese area was moved 4 times/pen during the rearing cycle. Geese were fed ad libitum with the same organic diet. The length of the rearing cycle was 140 days.

### Observers and training

Two observers were involved in the direct behavioral observations. The main observer (Observer 1) was a senior researcher with experience in poultry behavior, while the nonexpert observer (Observer 2) was a student in animal science who was familiar with farm animals but had no experience in formal behavioral observations. Observer 2 was trained by the main observer on poultry behaviors, on the ethogram prepared for geese, and on behavioral observation techniques. During the training, particular attention was given to the explanation of the terminologies of behavior used in the ethogram to clarify the meaning of each behavior reported. The training lasted approximately 20 days and included field observation sessions. By the end of the training period, an excellent level of interobserver agreement (ICC > 0.75%) was achieved.

### Behavioral observations

At 120 days of age, 10 out of the 25 geese in each pen and each replicate (*N* = 40) were randomly selected, weighed (4,200 ± 40 g), and individually marked with different colors by colored sticks for zootechnical use. On the same day, the different pens were inspected during the morning (9:00–9:30 AM) and the afternoon (15:00–15:30 PM) to establish the position and dimension of the geese area. For each pen, a geese area consisting of 300 m^2^ was identified. Then, a video camera (BASLER, ac A 1300–60 gc) was set up for each pen and oriented toward the geese area. The cameras were placed 2 m above the ground such that the field of view was a 20 × 10 m area/camera. In this way, it was possible to cover a total of 200 m^2^ corresponding to most of the geese area except for a blind spot of approximately 100 m^2^. Behavioral observations were recorded ten days later, i.e., during the last week of the rearing cycle (130 days of age), using the ethogram described in Table 5.

**Table 5:** Ethogram of recorded behaviors of geese.

Behavior	Description
Walking	The goose moves more than three steps
Resting	The goose presents its body in line with the ground with an erect head and open eyes
Roosting	The goose is in a lying position with the ventral body region in contact with the floor
Foraging	The goose presents its head down and beaks in contact with the grass
Feeding	The goose pecks inside the feeder
Drinking	The goose pecks the drinker
Getting wet	The goose bathes in the water tub
Self-grooming	The goose uses its beak to clean the feathers of the wings and the body
Aggression	The goose interacts through aggressive behaviors, such as pecking and blowing; usually ends with one goose running away
Flapping wings	The goose flaps its wings vigorously while keeping the neck and legs tense
Allo-grooming	The goose uses its beak to clean the feathers of the wings and the body of another conspecific
Squawking	The goose squawks
Wagging tail	The goose makes rapid movements with its tail to the right and to left
Neck forward	The goose extends its neck parallel to the ground
Neck up	The goose extends its neck perpendicular to the ground
Shaking head	The goose makes rapid movements with the head to the right and to left
Stretching	The goose makes a slow extension of some parts of the body (mainly wings and legs)
Panting	The goose shows fast, labored breathing with an opened beak

Two 30 min sessions per day were performed as reported above. Each session was replicated in the two different pens for each agroforestry system (AO and V). The behavioral data of each session were collected in two ways: (i) direct observation in the field and compilation of the collection forms (i.e., behavior observation form, BOF; Supplementary Fig. S1), and (ii) indirect observations of videotapes and analysis by a computerized system (Noldus Technology, Wageningen, The Netherlands).

Before each session of observation, the camera was turned on, and the two observers were positioned approximately 5 m behind the camera at a distance of approximately 2 m from each other (e.g., Fig. 3d). To allow the geese to get used to the observers’ presence and minimize their effects, the observers were located approximately 10 m from the geese area and waited 10 min before recording the geese's behavior. Consequently, the first 10 min of the video were discarded. Then, observers started to write down the behaviors of the marked geese on the BOF (e.g., Supplementary Fig. S1) by focal subgroup sampling^42^. Thus, all occurrences of the specified behaviors were recorded (as events) for each of the 10 selected geese in the pen during the 30 min session. All the selected geese were continuously visible throughout the session. The behavior of each goose was recorded for 60 min, and a total of 40 geese were observed. The data collected during direct observations by the main observer were used to verify the content and construct validity of the BOF, while those collected by Observer 2 were used to determine the interobserver agreement.

Approximately two months after the field observations, the main observer analyzed the videos of each session by continuous focal animal sampling using Observer XT software (Noldus, Technology, Wageningen, The Netherlands). The same ethogram of the BOF (e.g., Table 5) was used, and all the behaviors were recorded as events. The frequencies obtained by the computerized system were used to verify the criterion validity of the BOF.

### Statistical analysis

The different statistical approaches used to analyze the data are summarized in Supplementary Table S4.

First, the frequencies of the behaviors included in the BOF were presented using descriptive statistics. The events were expressed as absolute frequencies, as all animals were always visible for the entire observation period. Then, several statistical methods were used to test different aspects of the validation process, including both reliability and validity. The intraobserver reliability indicates the agreement between multiple people independently rating the same individual^43^, and it was evaluated by the two-way mixed model ICC applied to single measurements ^14–16^. ICC values were interpreted as poor (ICC < 0.40), fair (0.40 ≤ ICC < 0.60), good (0.60 ≤ ICC < 0.75), and excellent (ICC ≥ 0.75)^16,44^. Each ICC was followed by its 95% confidence interval (CI) and by the *p* value of the *F* test. The frequencies recorded by the two observers were also compared by the Wilcoxon signed-rank test.

The validity of the BOF was verified using the data collected from the main observer. Content validity expresses the degree to which an instrument is an adequate reflection of the construct to be measured^45,46^. The ethogram of the BOF should indicate all behaviors of interest observed in geese and include its broadest behavioral traits. Therefore, a PCA was conducted to extract the latent dimensions of the behavioral variables included in the BOF and verify whether these could indicate reliable broad behavioral traits of the geese. The behavioral variables were included in PCA as the mean of the frequencies collected during the morning and afternoon and after inspection of the correlation matrix and communalities^47^. PCs showing eigenvalues greater than 1 were retained and rotated with the Varimax method and Kaiser Normalization^48,49^. Only factor loadings with an absolute value greater than 0.5 were interpreted (corresponding to 25% variance explained). Cronbach’s alpha assessed the reliability of the PCs, and values > 0.7 were considered acceptable^50,51^. Finally, corresponding PC scores were calculated using the regression method^47,52^.

Criterion validity investigates the degree to which the instrument is an adequate reflection of a gold standard^15,45^. Thus, the correlations between the frequencies collected by the BOF and those recorded by the computerized system (treated as the gold standard measure) were calculated using the Spearman rank correlation coefficient (ρ). The correlation was considered poor if ρ < │0.3│, medium if │0.3│ ≤ ρ < │0.5│, and large if ρ ≥ │0.5│^15^. The two frequencies were compared by the Wilcoxon signed-rank test.

Finally, the BOFs recorded by the main observer were used to check whether the behavior of the geese changed during the day and whether the behavior was influenced by the agroforestry system. The behavioral variables were analyzed using generalized linear models (GLM) with a negative binomial distribution and a log link, as they showed a Poisson distribution and considerable overdispersion. Aggression and panting were infrequently recorded during sampling and were thus excluded from analysis. Geese were included in the GLM as subjects, and time was included as a within-subject effect. No behaviors were significantly affected by the replicate (*p* > 0.05). Each GLM evaluated the main effect of time (2 levels: morning and afternoon) and agroforestry systems (2 levels: AO and V). These analyses also verify the responsiveness over time (the ability of an instrument to detect change over time when change has occurred) and the construct validity (the degree to which the concept measured behaves as expected in relation to “known groups”) of the BOF^16,45,46^.

Analyses were performed with SPSS software, version 25 (SPSS Inc., Chicago, IL). P values of ≤ 0.05 were considered to be statistically significant; however, trends between *p* > 0.05 and *p* < 0.10 are also presented.

### Sample size

The a priori calculation of the required sample size was based on the analyses aimed at evaluating the effect of time and agroforestry system on geese's behavior. G*Power software (ver. 3.1.9.4) was used^53^. An F test for two-way mixed ANOVA including two groups (AO and V) and two repeated measurements (i.e., times) was envisaged. Setting α = 0.05, with a medium effect size (f = 0.25) and a power (1 − β) = 0.85, the minimum number of geese needed for evaluation was 38. This number was approximated to 40 (*n* = 20/system) to consider the attrition rate. As ten geese could be evaluated for each pen, two replicates were planned. The number expected for these analyses also satisfied the sample sizes required to observe a given interobserver agreement based on ICC procedures, as previously reported^15,16^. Indeed, 22 specimens were required by setting a 90% confidence interval of width Wk = 0.2, assuming a planning value of ICC (ICCplan) = 0.85, and k = 2 observers.

## Supplementary Information

Supplementary Information.

## Data Availability

The datasets generated during and analyzed during the current study are included in this published article (and its Supplementary Information files) and are available from the corresponding author upon reasonable request.
