# Supplementary material for: Validation of a behavior observation form for geese reared in agroforestry systems
Source: Sci Rep. 2022 Sep 7;12:15152. doi: 10.1038/s41598-022-18070-6 (PMC9452672; doi:10.1038/s41598-022-18070-6)
Supplement: Supplementary file 1 — Supplementary Information. [file 41598_2022_18070_MOESM1_ESM.pdf]

Supplementary materials

Supplementary Table S1. Mean and standard deviation (s.d.) of frequencies recorded by the behaviour observation form in field and the 'gold standard' measure (i.e. Noldus) regardless of time and agroforestry system (n=20).

Behavior	Field observations		Noldus analysis		P value *
	Mean	s.d.	Mean	s.d.	
Grass	3.93	4.58	4.50	4.85	<0.001
Drink	1.23	1.53	1.73	1.99	<0.001
Walk	3.35	3.72	4.07	4.09	<0.001
Rest	2.50	1.06	3.22	1.14	<0.001
Roost	1.42	2.50	1.68	2.71	0.004
Grooming	1.90	2.56	2.45	2.83	<0.001
Attack	0.02	0.16	0.05	0.32	0.317
Flap wings	1.18	1.87	1.50	2.20	0.001
Allogrooming	0.10	0.50	0.18	0.84	0.180
Neck forward	0.27	0.45	0.48	0.82	0.005
Quaking	0.05	0.22	1.08	0.94	<0.001
Get wet	0.77	2.17	0.95	2.35	0.008
Neck up	0.02	0.16	0.33	0.62	0.003
Panting	0.02	0.16	0.05	0.32	0.317
Stretching	0.02	0.16	0.40	0.71	0.002
Feed	0.18	0.50	0.25	0.71	0.083
Wag tail	0.02	0.16	0.55	0.75	<0.001
Shake head	0.18	0.50	0.48	0.82	<0.001

* Wilcoxon signed rank test.

Supplementary Table S2. Significance of the main effects evaluated by the Generalized Linear Models.

**Behavioural variable**	**Time**	**Agroforestry system**
Walking	<0.001	<0.001
Resting	<0.001	<0.001
Roosting	<0.001	<0.001
Foraging	<0.001	0.398
Feeding	0.076	-[a]
Drinking	0.048	<0.001
Getting wet	0.929	0.479
Grooming	<0.001	0.099
Aggression	-[b]	-[b]
Flapping wings	0.393	0.137
Allo-grooming	-[a]	0.589
Squawking	0.848	0.729
Wagging tail	0.246	0.739
Neck forward	0.021	0.465
Neck up	0.277	0.317
Shaking head	0.142	0.282
Stretching	0.494	0.036
Panting	-[b]	-[b]

-[a] unable to compute due to numerical problem
-[b] behaviours recorded infrequently and excluded from analysis

Supplementary Table S3. Minimum and maximum temperature and average relative humidity during the period of behaviural evaluation.

**Month**	**Temperature (°C)**		**Humidity (%)**
	**Min**	**Max**	
**June**	15.7	31.1	58.3
**July**	18.1	32.0	58.6
**August**	18.0	32.5	61.8

Supplementary Table S4. Statistical methods used for validation of the behavior observation form (BOF) for geese.

Type of analysis	Description	Statistical test
Distribution of the frequencies	Distribution of the frequencies recorded by the two observers	Descriptive analysis
	Differences between the frequencies recorded by the two observers	Wilcoxon signed-rank test
Inter-observer reliability	Agreement among the two blinded observers	Intraclass correlation coefficient
Content validity	Extraction of the latent dimensions that describe reliable behavioral traits of geese.	Principal Component Analysis and Cronbach's alpha
Criterion validity	Correlation between the frequencies obtained in the field by the BOF (focal subgroup sampling) and the 'gold standard' measure (focal animal sampling by computerized system)	Spearman rank correlation coefficient and Wilcoxon signed-rank test
Construct validity and responsiveness	Differences between the frequencies recorded by the main observer on the same geese at two different times (morning vs afternoon). The hypothesis was that the geese's behavior evaluated by the BOF changes according with the time of the day (responsiveness over time).	Negative binomial distribution with log link function
	Differences between the frequencies recorded by the main observer in geese reared in two agroforestry systems (vineyard vs orchard). The hypothesis was that the geese's behavior evaluated by the BOF changes according with the agroforestry system (differentiation by "known groups").	

Supplementary Figure S1. Behavior Observation Form (BOF) used to direct observation in the field.

TRIAL NAME__________________

DATE________________________

TIME (AM/PM)____________________

OBSERVER_________________

Behaviours	ID Geese									
	1	2	3	4	5	6	7	8	9	10
Walking										
Resting										
Roosting										
Foraging										
Feeding										
Drinking										
Getting wet										
Self-grooming										
Aggression										
Flapping wings										
Allo-grooming										
Squawking										
Wagging tail										
Neck forward										
Neck up										
Shaking head										
Stretching										
Panting										

Note ____
